# Market research of infant formula milks in Spain

**DOI:** 10.1002/fsn3.3712

**Published:** 2023-09-27

**Authors:** Irene Albertos, Janira Romero, Alicia Piqueras‐Picón, Sara García, María López, José María Jiménez, María José Castro Alija

**Affiliations:** ^1^ Recognized Research Group: Assessment and Multidisciplinary Intervention in Health Care and Sustainable Lifestyles of the University of Valladolid Valladolid Spain; ^2^ Catholic University of Avila, (UCAV) Avila Spain

**Keywords:** breast milk, formula milk, infant feeding, market research, nutritional criteria

## Abstract

There are circumstances in which breastfeeding is not possible and formula milk feeding has to be resorted to. In these cases, it is difficult to choose among the multitude of brands on the market for infant feeding. Although the composition of formula milk is largely regulated by legislation, there are certain nutrients whose presence in breast milk is beneficial. This circumstance will help us to establish some criteria to choose formula milk for a healthy infant. Among all the formula milks which can be found in different shops (pharmacies, supermarkets, etc.), market research has been carried out based on the nutritional criteria: the lipid and protein profile, certain carbohydrates, amino acids, and vitamins, as well as the presence of nucleotides, prebiotics, and probiotics. Based on these results, it can be established which formula milk we would give to a healthy infant. Thus, within the analyzed formula milks, it has been concluded that the most complete milk in this would be formula milk D and the least formula milk J. Although more studies are needed to confirm this, it is foreseeable that, as the scientific evidence is greater, the legislation will be updated and considering these nutrients for a correct formula milk.

## Graphical abstract

The maximum possible similarity of formula milks to breast milk in terms of components is important. The most complete formula milk found on the Spanish market, according to the needs of the infant is formula D. Formula milks must be safe and effective for the infant and comply with the established legislation.

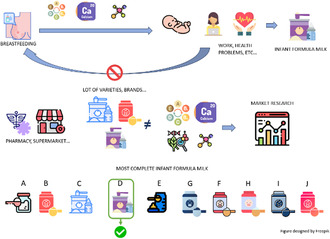

## 1 INTRODUCTION

The WHO recommendation regarding breastfeeding is to maintain exclusive breastfeeding up to 6 months, and together with complementary feeding, up to 2 years of age (WHO, 2006, 2021). Despite this, worldwide it is estimated that the percentage of children under 6 months exclusively breastfed does not reach 40% (Machado et al., 2014).

In Spain, according to the Health Survey 2017 of the Institute of Statistics (INE, 2017), it gives the following data in relation to breastfeeding: during the first 6 weeks, 73.9% of babies are fed with breast milk. Then, it decreases to 63.9% when they reach 3 months, until reaching 39% when they are 6 months old. It follows from these data that as the months pass, breastfeeding decreases. Sociodemographic factors could influence, but in this survey, it was seen that the socioeconomic level did not affect the type of breastfeeding too much.

However, these data are compared with the data obtained from the previous survey conducted by the Institute of Statistics, the Health Survey 2011–2012 (INE, 2013), and it can be observed how there has been an increase in recent years of breastfeeding compared to artificial at all stages of lactation.

At the European level, Spain is not among the countries with the highest breastfeeding rates. The highest rates measured at 6 months of the baby, occurred in Norway (71%), Sweden (61%), and Germany (57%; Theurich et al., 2019). One of the possible causes is that these countries that have the highest rates of breastfeeding usually have social and labor policies that favor it. They also usually have more information and awareness of maternal prolactin.

Breast milk has some characteristics that make it special, so it is very difficult to imitate. Its composition changes throughout the day, having a higher fat content at the end of the day. It also varies throughout breastfeeding, during the first days of breastfeeding, when the so‐called colostrum is generated, it has a higher content of water, proteins, mineral salts, and immunoglobulins, and it adapts to the nutritional needs of the child as he grows (Martín Martínez, 2005). Then, from the age of 4 weeks, when the milk is considered mature, the composition will hardly undergo variations, maintaining a stable amount of carbohydrates, proteins, and lipids. In the case of proteins, there are some recent studies that indicate that their concentration in milk decreases slightly until the first year, but it seems that then they begin to increase (Suárez Rodríguez et al., 2020). In breast milk, its components are forming three different fractions: lipid fraction, in the form of an emulsion with fat globules, a second fraction containing proteins in suspension forming micelles, and finally, the phase containing the water‐soluble components in solution (Vass et al., 2019).

In the lipid fraction, there are fatty acids, cholesterol, and fat‐soluble vitamins (A, D, and E). Lipids constitute the main source of energy, and as we have seen, they are found in a high proportion, more than 4%. It is important to highlight that it is arranged in the form of an emulsion of fat globules (milk fat globule membrane [MFGM]), and that the membranes of these globules are formed by various substances, among which phospholipids highlight, such as gangliosides, which contribute to the development of the central nervous system; and in addition, it seems that they also intervene in the regulation of the immune system and even in the intestinal microbiota of the infant (Claumarchirant et al., 2017). Polyunsaturated fatty acids, arachidonic acid, stands out, fundamental in neuronal development (Rivero Urgell et al., 2005), which has short‐chain fatty acids and esters, to which an important bactericidal activity is attributed. In addition, the presence of β‐palmitate is important since it has beneficial effects on fatty acid metabolism, increases calcium absorption, and improves stool consistency by making it softer (Mancini et al., 2015).

Casein forms micelles together with calcium and phosphorus, thus also allowing the incorporation of these minerals. Breast milk has less casein than cow's milk, which makes it more digestible. Among the whey proteins, alpha‐lactalbumin is necessary for the synthesis of lactose. Lactoferrin has an essential role; even though breast milk is poor in iron, lactoferrin promotes the absorption of iron in the intestine, thus increasing its bioavailability (Helman et al., 2019). The needs of the infant in relation to iron are covered with breast milk until the age of 6 months, but after that, food supplementation is necessary.

The presence of amino acids also plays a very important role in breast milk. Amino acids such as tryptophan in the infant behave as an essential amino acid; it is a precursor of serotonin that intervenes among other things in the regulation of sleep (Bravo et al., 2018). Taurine is another abundant amino acid in breast milk and its presence is related to the better absorption of fats and with the correct development of the central nervous system and that of the retina (Almeida et al., 2021). Another amino acid present in breast milk and with great relevance in multiple metabolic processes is carnitine (Rivero Urgell et al., 2005).

Likewise, breast milk also contains nucleotides in its composition, essential nutrients in the modulation of the immune system (Rivero Urgell et al., 2005).

Choline (water‐soluble vitamin of group B) is another essential nutrient that is present and is fundamental in the cognitive development of the infant (Wallace et al., 2018).

Among the enzymes, the high content of lipase in breast milk helps lipids to be more easily digested. It also has a high concentration of lysozyme (unlike cow's milk) that has a bactericidal action at the intestinal level (Macías et al., 2006). Breast milk confers immunity to the infant, thanks to its richness in lysozyme and immunoglobulins.

A multitude of minerals are also found in breast milk (calcium, potassium, magnesium, selenium, zinc, etc.). Its concentration is adapted to the metabolic capacity and nutritional needs of the infant. For example, in the case of calcium and phosphorus, the ratio in breast milk is the ideal one as its absorption is optimal (Lawrence, 2022).

The composition of breast milk has been used as the “gold standard” to search for the optimal formulation of an artificial milk. The last update of the Reference Dietary Intakes (RDI) was carried out in 2019 by the Scientific Committee of the Spanish Agency for Food Safety and Nutrition (AESAN) (AESAN, 2019). When establishing criteria, the decision‐making algorithms of the FESNAD were applied.

To establish the RDI of macronutrients and energy in the population of our study, from 0 to 12 months, we use the values established in the Food Composition Tables of Moreiras et al. (2016) and those of the Academies of Sciences Engineering Medicine (The Academies of Sciences Engineering Medicine, 2023), since they are the only ones from which data can be obtained in this age group.

Regarding macronutrients, the energy, protein, and carbohydrate needs increase as the baby grows, being higher at 12 months than at 6; however, lipids remain constant.

As for micronutrients, high amounts of certain vitamins such as vitamin A, D, E, niacin, and folate are required. On the other hand, the requirements of niacin, vitamin B12, and vitamin K practically double from 6 to 12 months. Of the minerals, those whose needs are increased significantly as the baby's growth progresses are sodium, potassium, magnesium, chloride, chromium, iron, and copper. The case of iron acquires special importance because there is a risk of deficiency from the age of 6 months in cases where the infant is only breastfed.

As stipulated by the Spanish Agency for Food Safety and Nutrition (AESAN), it may be called “milk,” when it is made entirely from cow's or goat's milk proteins, while it is called “prepared,” liquid food products intended to meet the nutritional needs of healthy infants (and are not made from cow's or goat's milk proteins).

Both milks and preparations are in turn classified into two types, depending on the age of the infant:

1. Initial: intended to completely cover the nutritional needs of the infant during the first months of life until the introduction of complementary feeding (up to 6 months).
2. Continuation: intended for infants (from 6 to 12 months) to whom complementary feeding is already introduced, constituting the main liquid element of their diet.

The legal requirements regarding the composition and the information that must appear on the packaging of infant formula milks are regulated by Regulation (EU) No 609/2013 and Delegated Regulation (EU) 2016/127. Both are already mandatory, except for formulations made from protein hydrolysates, which did not become mandatory until February 22, 2022. Until this date, the provisions of Royal Decree 867/2008 continued to be applied, unless otherwise repealed. There are also two other regulations: Delegated Regulation (EU) 2018/561 which refers to the protein requirements of follow‐on formulas and Delegated Regulation (EU) 2019/828, which regulates the requirements for vitamin D and erucic acid. Regulation (EU) No. 609/2013 contains a list of substances that can be added to milk or formula preparations called the “Union List.” The substances will belong to the following categories: vitamins, minerals, amino acids, carnitine and taurine, nucleotides, choline, and inositol. The substance is specified and in what form it may appear, although the amount is not specified. For example, vitamin A may appear as retinol, retinyl acetate, or retinyl propionate but not as beta‐carotene.

Subsequently, Delegated Regulation (EU) 2016/127, supplementing Regulation (EU) No 609/2013, establishes and updates the specific composition and information requirements applicable to milk and infant formula. It addresses all the aspects that formula milks must comply with at a regulatory level.

Therefore, the general requirements are:

1. It is mandatory that the quantities of each component or added substance that is part of the milk formula must be specified.
2. The quantities or the minimum and maximum limits of many of the macro and micronutrients are fixed by EFSA. Being mandatory in the starter milks, the specification of the amount of choline, inositol, and carnitine.
3. Infant formula may not contain any “nutritional” or “health properties” statements.
4. Advertising in places of sale, distribution of samples or any other method that is aimed directly at the consumer to encourage sales of milk and infant formula is prohibited.

The main objective of this study is to carry out an analysis of the formula milks currently on the Spanish market for healthy infants, based on their content in certain nutrients and ingredients that have proven to have beneficial properties for the development of the baby. With this, we could establish some criteria, which regardless of other factors, such as price, could help us to choose which would be the most complete and adequate formula milk for a healthy infant.

## 2 MATERIALS AND METHODS

### 2.1 Formula milks selection

Formula milks available in the Spanish market were examined. To get as close as possible to the real conditions, a search was carried out of all the formula milks for healthy infants available on the market that anyone could find in their usual trade. Therefore, we proceeded to visit three types of establishments, all located in the province of Madrid: pharmacies (7), parapharmacy section (3), and supermarkets (Carrefour, E.Leclerc, Hipercor, Dia, AhorraMas and Alcampo).

When selecting formula milks, all milks categorized as “Food for special medical uses” were excluded and we focused on the milks that are objects of our study: infant milks, that is, babies up to 12 months old and without pathology that require special feeding. They are also classified according to the age of the infant: starting milks (from 0 to 6 months) and follow‐up milks: intended for infants (from 6 to 12 months).

In short, the sample with which we carried out the study was composed of all the brands of formula milks that were seen in the 16 establishments visited, considering this selection as a representative sample of what anyone could find in the different shops. There are a total of 10 different trademarks of cow's milk from which both the initial and the continuation formulations will be chosen. As for obtaining the nutritional information and composition of each formula milk, it is obtained from the outside of the corresponding containers.

The formula milks studied were anonymized to compare its quality. The samples were named as below.

1. Formula milk A (Start and Continuation)
2. Formula milk B (Start and Continuation)
3. Formula milk C (Start and Continuation)
4. Formula milk D (Start and Continuation)
5. Formula milk E (Start and Continuation)
6. Formula milk F (Start and Continuation)
7. Formula milk G (Start and Continuation)
8. Formula milk H (Start and Continuation)
9. Formula milk I (Start and Continuation)
10. Formula milk J (Start and Continuation).

### 2.2 Nutritional components object of study

The nutrients and ingredients studied were the following:

1. Fats: Arachidonic acid (AA), MFGM (milk fat globule membrane), β palmitate (milk fat), and absence of palm oil. Although some formulas did not contain palm oil, we also considered if they had other saturated fat such as coconut oil. In addition, the ratio of linoleic/α‐linolenic essential fatty acids were also assessed.
2. Carbohydrates: Lactose/Sugars, Inositol.
3. Proteins: Seroproteins/Casein.
4. Amino acids: Taurine, L tryptophan, L carnitine (only accepted in starter formulas).
5. Nucleotides.
6. Vitamins: Choline.
7. Prebiotics: Galactooligosaccharides (GOS), Inulin, 2′fucosyl‐lactose (2FL), Polydextrose.
8. Probiotics.

At first, both the starting and the continuation formulations were chosen, but when looking at the nutritional information of the different formula milks, the first thing that was observed is that when any of the nutrient's object of our study was present in the starting formula of a certain milk, it was also present in its corresponding continuation formula, and vice versa, so we proceeded to perform this analysis jointly for both formulations. Only the information of the two formulations had to be collected separately when obtaining the quantitative data necessary to assess the lipid profile of essential fatty acids (linoleic and α‐linolenic), the amount of lactose with respect to the total sugar content and the ratio between seroproteins/casein.

## 3 RESULTS

### 3.1 Special interest nutrients presence

Directives of the European Commission, the European Society for Pediatric Gastroenterology Hepatology and Nutrition (ESPGAN) and other organizations such as the Spanish Association of Pediatrics and the Spanish Agency for Food Safety and Nutrition (AESAN) establish the nutrients in formula milks.

Nutrients analyzed in the 10 commercial brands of formula milks were found in Table 1.

**TABLE 1:** Special interest nutrients' presence in commercial formula milks (start and continuation formula milks).

	Formula milk A	Formula milk B	Formula milk C	Formula milk D	Formula milk E	Formula milk F	Formula milk G	Formula milk H	Formula milk I	Formula milk J
Lipids										
Arquid acid. (AA)	x	x	x	x	x	x	x	x	x	x
MFGM	x			x				x	x	
β palmitate (milk fat)			x	x				x	x	
Without palm oil		x (coconut)^a^	x (coconut)^a^	x	x	x		x (coconut)^a^	x (coconut)^a^	
Linoleic/⟨‐linolenic (⌉6/⌉3)	x	x	x	x	x	x	x	x	x	x
Carbohydrates										
Lactose/sugars	Unespecified	x	x	x	x	x	Unespecified	x	x	Unespecified
Inositol	x	x	x	x	x	x	x	x	x	x
Proteins										
Seroproteins/Casein	Unespecified	x	x	x	Unespecified	Unespecified	Unespecified	x	x	x
Amioacids										
Taurin	x	x	x	x	x	x	x	x	x	
L triptofan	x	x		x						
L carnitin (starter milks)	x	x	x	x	x	x	x	x	x	
Nucleotids			x	x		x	x	x	x	
Vitamin										
Choline	x	x	x	x	x	x	x	x	x	x
Prebiotics										
Galactooligosaccharides (GOS)	x	x	x	x			x	x	x	x
Inulin				x						
2′fucosyl‐lactose (2′FL)	x		x	x		x			x	
Polydextrose										
Probiotics			x	x		x				

^a^ Although it does not contain palm oil, it contains another saturated oil (coconut oil).

### 3.2 Lipids (arachidonic acid, MFGM, β‐palmitate, and palm oil)

Table 1 showed that three of the milk brands (Formula milk D, Formula milk H, and Formula milk I) contained arachidonic acid, MFGM, and β palmitate in their composition, and they did not include palm oil. However, in the last two, instead of palm oil, they had used another saturated fat, coconut.

#### 3.2.1 Essential fatty acid content: linoleic acid (ω‐6) and α‐linolenic acid (ω‐3)

**FIGURE 1:**
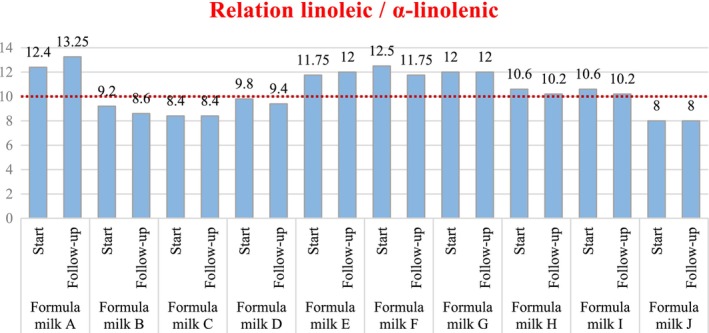
Linoleic (‐6) acid/‐linolenic (‐3) in formula milks relation.

All formula milks contained the essential fatty acids linoleic (ω‐6) and α‐linolenic (ω‐3) in their composition, but it was important to see in what proportion they included them. Since the linoleic acid/α‐linolenic acid ratio in breast milk is approximately 10/1, considering this ratio, in which portion these fatty acids were found in the different formula milks. Breast milk was established as the reference ratio (Figure 1):

The formula milks that moved the furthest away from this optimal relationship were Formula milk A (start and follow‐up), Formula milk F (start), and Formula milk J (start and follow‐up), while the ones that came closest were Formula milk D (start), Formula milk H (follow‐up), and Formula milk I (follow‐up).

As it had been verified in the analyzed milks and as expected, they all contained linoleic and α‐linolenic acid in their composition. What varied between some milks and others was the existing relationship between the two taking as a reference the existing relationship in breast milk, which would be the ideal one and the one that will always be tried to be emulated.

If the linoleic and alpha‐linolenic values of the different formula milks were observed (Table 2), it would be seen that the alpha‐linolenic values hardly vary, while the linoleic was the one in which the most difference could be found comparing the different milks, both by excess and by default. The amount of this nutrient was the most conditioning parameter to achieve the optimal ratio between linoleic acid (ω‐6)/α‐linolenic acid (ω‐3).

**TABLE 2:** Linoleic (ω‐6)/α‐linolenic (ω‐3) in formula milks relation.

	Linoleic (ω‐6) (g/100 mL)	α‐linolenic (ω‐3) (g/100 mL)	Linoleic/α‐linolenic
Formula milk A			
Start	0.62	0.05	12.4/1
Follow‐up	0.53	0.04	13.25/1
Formula milk B			
Start	0.46	0.05	9.2/1
Follow‐up	0.43	0.05	8.6/1
Formula milk C			
Start	0.42	0.05	8.4/1
Follow‐up	0.42	0.05	8.4/1
Formula milk D			
Start	0.49	0.05	9.8/1
Follow‐up	0.47	0.05	9.4/1
Formula milk E			
Start	0.47	0.04	11.75/1
Follow‐up	0.48	0.04	12/1
Formula milk F			
Start	0.50	0.04	12.5/1
Follow‐up	0.47	0.04	11.75/1
Formula milk G			
Start	0.60	0.05	12/1
Follow‐up	0.60	0.05	12/1
Formula milk H			
Start	0.53	0.05	10.6/1
Follow‐up	0.51	0.05	10.2/1
Formula milk I			
Start	0.53	0.05	10.6/1
Follow‐up	0.51	0.05	10.2/1
Formula milk J			
Start	0.40	0.05	8/1
Follow‐up	0.40	0.05	8/1

### 3.3 Carbohydrates (lactose and inositol)

#### 3.3.1 Lactose and total sugar content

Lactose is important because in addition to providing energy, it causes the intestinal pH acidification, thus favoring the absorption of minerals such as calcium, and the growth of some bacteria beneficial to the intestinal microflora, such as *Lactobacillus bifidus*. It is also involved in the formation of galactocerebrosides, fundamental for the baby's brain development. Therefore, the lactose content in breast milk is very high and is found in an approximate amount of 6–6.5 g/100 mL (Lawrence, 2022), this being the majority sugar.

Based on this, and always taking breast milk as a reference, the lactose content should be specified in formula milks in all cases, and that this should appear as the majority compared to total sugars.

The lactose content was not available in two of the milks analyzed (Formula milk A and Formula milk G) and only one of the formulations (Formula milk D ‐continuation) contained an amount of lactose that is within the established reference range of breast milk (Table 3). In relation to the percentage of lactose with respect to the content of total sugars, it could be considered that lactose is the majority sugar in all formula milks.

**TABLE 3:** Total lactose content respect total sugar content and ratio of seroproteins to casein.

	Lactose (g/100 mL)	Total sugars (g/100 mL)	Seroprotein/casein (g/100 mL)	Seroprotein/casein (%)
Formula milk A				
Start	7.01	7.3	0.7/0.7	(50%/50%)
Follow‐up	7.8	8.1	0.5/0.8	(38%/62%)
Formula milk C				
Start	7.1	7.1	0.74/0.49	(60%/40%)
Follow‐up	5.5	5.6	0.72/0.59	(55%/45%)
Formula milk D				
Start	6.8	7	0.7/0.5	(58%/42%)
Follow‐up	6.3	6.4	0.6/0.6	(50%/50%)
Formula milk E				
Start	8.1	8.1		
Follow‐up	‐	5.5		
Formula milk F				
Start	7.6	7.6		
Follow‐up	8.4	8.4		
Formula milk H				
Start	6.8	7	0.5/0.8	(38%/62%)
Follow‐up	5.3	5.6	0.5/0.9	(36%/64%)
Formula milk I				
Start	6.8	6.9	0.5/0.8	(38%/62%)
Follow‐up	5.3	5.6	0.5/0.9	(36%/64%)
Formula milk J				
Start	6.9	7	−/−	−/−
Follow‐up	7	7.2	0.6/0.9	(40%/60%)

One of the things that could draw our attention from the results obtained is that, in many of the milks, the continuation formula had a lower sugar content than the initial formula, and this was contradictory when precisely the carbohydrate needs increased as the baby grew, the requirements being higher at 12 than at 6 months.

#### 3.3.2 Inositol content

Inositol is a nutrient that can be found in all the formula milks analyzed, so this would not be a criterion that we could use a priori to compare the different formula milks.

### 3.4 Proteins

#### 3.4.1 Ratio of seroproteins to casein

In four of the formulas (Formula milk A, Formula milk E, Formula milk F, and Formula milk G), the seroprotein and casein values were not specified. Of the rest of the formulas in which they were specified, the following data were collected (Table 3).

Of the formula milks analyzed, these values were not expressed in four of them, of the remaining six, only two initial formulations (Formula milk C and Formula milk D) had an optimal ratio between seroproteins/casein of (60%/40%), while in the continuation formulations, four of them had a ratio very far from the one we had established as a reference and only one (Formula milk D) that contained an adequate ratio of seroproteins/casein.

#### 3.4.2 Amino acid content. (taurine and L‐carnitine, L‐tryptophan)

The amino acids taurine and L‐carnitine appeared in all formulas except one of the formulations (Formula milk J), whereas L‐tryptophan only appeared in three of the formulations (Formula milk A, Formula milk B, and Formula milk D). Further, Formula milk J being the only one that did not contain any of the amino acids.

### 3.5 Nucleotide content

Of the 10 formula milks analyzed, six contained nucleotides in their composition, whereas in four of them (Formula milk A, Formula milk B, Formula milk E, and Formula milk J), they were not present.

#### 3.5.1 Choline content

Like inositol, it was a nutrient that was present in all the formula milks analyzed, so we could not use it as a criterion to compare the different formula milks.

### 3.6 Prebiotics and probiotics content

All the formulations except one (Formula milk E) had at least one prebiotic, being Formula milk D the milk that carried the most prebiotics (GOS, Inulin, and 2FL). The presence of probiotics was, however, less widespread among formula milks, and only three of the milks analyzed carry them (Formula milk C, Formula milk D, and Formula milk F). The probiotic species that were usually used in formula milks were of the genus *Bifidobacterium* and *Lactobacillus* (*B. infantis, B. longum, L. casei* subsp. *Rhamnosus*, *and L. reuteri*; De Almagro Garcia et al., 2017), since it was known that bacteria of the genus *Bifidobacterium* predominate in the microbiota of breast‐fed infants (Schack‐Nielsen & Michaelsen, 2007).

## 4 DISCUSSION

All formula milks on the market comply with what is established by legislation regarding specific composition and information requirements. Some general requirements are established such as the obligation to specify the quantities of each nutrient, setting minimum and maximum limits for many of them; the prohibition of carrying any “nutritional declaration” or “healthy property”; as well as not being able to make direct advertising to the consumer that encourages the sales of these milks. More specific requirements are also established regarding the composition they must carry, in relation to the energy intake, macronutrients, and micronutrients. Based on this regulation, it can be considered that all formula milks should be the same or very similar, and there should not be so much variety on the market, but this is not the reality.

There is no doubt that no formula milk is and can never be better than breast milk, but the discussion arises when establishing nutritional parameters that help us define what would be the best composition for a formula milk, beyond the reference of breast milk. There are no publications or guidelines on this aspect that define these criteria. Barrio et al. (2015) tried to reach consensus and to know the opinion of a panel of experts in gastroenterology and child nutrition on certain nutritional aspects of infant formulas, such as the lipid profile established by the contribution of vegetable fats and beta‐palmitate; the use of hydrolyzed proteins; the percentage of lactose or that of other sugars such as glucose or dextrinomaltose; the role of DHA and arachidonic acid; taurine and lactoferrin supplementation and the use of prebiotics. The conclusion of this study was that the professional criterion when defining which of the nutrients would really bring benefits to the composition of formula milks was not unified.

Always taking breast milk as a reference and looking at those nutrients that the legislation does not yet contemplate as mandatory in formula milks, or for which there is no established criterion or limit amount, it can be seen how in this aspect differences could be established between some milks and others in terms of their composition. These nutrients have a very important role in the development of the infant:

### 4.1 Arachidonic acid (AA)

Although its addiction is not yet mandatory, recently in 2020, the European Academy of Pediatrics and the Child Health Foundation has published a report (Koletzko et al., 2020) assessing the need to include in formula milks, the acid arachidonic, on the grounds that in addition to being present in a significant amount in the breast milk, it contributed to neuronal development and the immune system (Salem Jr & Van Dael, 2020).

### 4.2 MFGM (milk fat globule membrane)

The arrangement of breast milk fat in the form of fat globule emulsion makes it more digestible and promotes the absorption of nutrients. In addition, forming part of the membranes of the fat globules are the phospholipids, most notably gangliosides, which are important because they contribute to the development of the central nervous system, to the regulation of the immune system and microbiota intestinal of the infant (Claumarchirant et al., 2017). For these reasons, those formula milks would have an added value that contain MFGM in their composition.

### 4.3 β palmitate

Breast milk fat is rich in β‐palmitate and effects are attributed to it being beneficial on the metabolism of fatty acids; it promotes the absorption of calcium and makes that the stool consistency is softer, preventing constipation (Mancini et al., 2015). In this point, it should be noted that what should not be present in formula milks is the palmitate that comes from palm oil.

### 4.4 Taurine

It is involved in the correct development of the central nervous system and that of the retina (Chesney et al., 1998), which is especially important for premature babies. In addition, it improves the absorption of fats.

### 4.5 Prebiotics and probiotics

They can be found in breast milk, and it is known that they contribute to the correct balance of the infant's microbiota. The gastrointestinal tract and the newborn's immune system are immature. It has been shown that the existence of a proper intestinal microbiota in the early stages of life helps to mature the system immune system (Spahn & Kucharzik, 2004). The intake of probiotics and prebiotics can favor the correct development of the intestinal microbiota.

Although the current trend is to use more and more except for this oil in formula milks, we can still see it among their ingredients. Sometimes, it appears masked under different names: palmolein, palm kernel oil, palm stearin, palm olein, or palm butter.

### 4.6 DHA

Although it is mandatory to add DHA in all formula milks, it is a nutrient that the manufacturer should call us with especially the attention on labeling.

It can be concluded that to assess the lipid profile of the different formula milks, the formula with a better lipid profile would be the one that has in its composition arachidonic acid, MFGM, β palmitate, and does not contain palm oil or other saturated oil, such as coconut and whose ratio of linoleic/α‐linolenic‐essential fatty acids is adequate (about 10/1). Among the formula milks analyzed, Formula milk D would be the one that would meet the most criteria.

Even though, as we have seen, the composition of infant formula milks must comply with the provisions of the legislation, every day we have more data and evidence regarding the benefits of the presence, or in some cases absence, of certain components that the legislation does not yet contemplate or on which no criteria have been established. This fact is what makes the manufacturing laboratories try to improve their formulas more and more, and thus be able to differentiate themselves from the rest of the formula milks on the market.

Although more studies are needed to confirm this, those formula milks that contain the nutrients and ingredients that we have already seen turn out to be beneficial will have an added value. Based on this, we could establish some preferences when choosing the formula milk that we would give to a healthy infant; so, within the formula milks that we have analyzed, the most complete milk in this sense would be Formula milk D and the one that is less complete would be Formula milk J.

From this study, we concluded that all infant formula milks currently available on the market were subject to specific legislation that makes them safe and effective for feeding infants, always considering them as an alternative to breastfeeding, which is the one that is recommended in the first place. There are nutrients whose presence in formula milks has been shown to be beneficial, although more confirmatory studies were needed as in the case of prebiotics and probiotics in which the types and the necessary doses with which concreted health benefits would be obtained are not yet defined. It is foreseeable that the legislation will be updated and contemplating these nutrients according to the scientific evidence will be greater. Moreover, the labeling of formula milks in general was difficult to read and sometimes it was not easy to find a certain nutrients or ingredients.

Sometimes, certain brands of milk used certain nutrients as a sales claim, although they were nutrients that must be added to all formula milks compulsorily because this is what the legislation has said as in the case of DHA.

## AUTHOR CONTRIBUTIONS

**Irene Albertos:** Conceptualization (equal); data curation (equal); formal analysis (equal); funding acquisition (equal); investigation (equal); methodology (equal); writing – review and editing (equal). **Janira Romero:** Writing – original draft (equal). **Alicia Piqueras‐Picón:** Conceptualization (equal); formal analysis (equal); writing – original draft (equal). **Sara García‐Villanueva:** Conceptualization (equal); investigation (equal); methodology (equal). **María López:** Data curation (equal); formal analysis (equal); funding acquisition (equal). **José María Jiménez:** Funding acquisition (equal); project administration (equal); resources (equal). **María José Castro‐Alija:** Conceptualization (equal); funding acquisition (equal); investigation (equal); validation (equal); writing – review and editing (equal).

## CONFLICT OF INTEREST STATEMENT

The authors declare no conflicts of interests.

## ETHICAL APPROVAL

This study does not involve any human or animal testing.

## Data Availability

The data that support the findings of this study are available on request from the corresponding author. The data are not publicly available due to privacy or ethical restrictions.
